# Benchmark Dose Approach to DNA and Liver Damage by Chlorpyrifos and Imidacloprid in Male Rats: The Protective Effect of a Clove-Oil-Based Nanoemulsion Loaded with Pomegranate Peel Extract

**DOI:** 10.3390/toxics11070569

**Published:** 2023-06-30

**Authors:** Alia Ahmed Abdel-Hamid Omar, Marwa Farouk Gad, Amel A. Refaie, Hemmat Mansour Abdelhafez, Abdel-Tawab H. Mossa

**Affiliations:** 1 Pesticide Chemistry Department, Chemical Industries Research Institute, National Research Centre, 33 El Bohouth Street (Former El Tahrir St.), Dokki, Giza 12622, Egypt; aliaahmed24@yahoo.com (A.A.A.-H.O.); marwagad280@yahoo.com (M.F.G.); dramelrefaie@yahoo.com (A.A.R.); 2 Cytochemistry and Histology, Zoology and Entomology Department, Faculty of Science (For Girls), Al-Azhar University, Cairo 11651, Egypt; hemmatmansour.sci.g@azhar.edu.eg

**Keywords:** insecticide, chlorpyrifos, imidacloprid, toxicity, oxidative stress, nanoemulsion, clove oil, pomegranate, liver, benchmark dose, rats

## Abstract

Pesticides are widely used around the world to increase crop production. They also have negative impacts on animals, humans, and the ecosystem. This is the first report evaluating a novel pomegranate-extract-loaded clove-oil-based nanoemulsion (PELCN) and its potential for reducing oxidative stress and DNA damage, as well as its hepatoprotective effects against imidacloprid (IM) and chlorpyrifos (CPF) toxicity in male rats. The benchmark dose (BMD) approach was also used to study the dose–response toxicity of IM and CPF. IM and CPF were administered daily for 28 days at doses of 14, 28, and 54 mg/kg body weight (bw) of IM and 1, 2, and 4 mg/kg bw of CPF via drinking water. The PELCN was administered orally at a dose of 50 mg/kg bw/day of pomegranate extract, 500 mg/kg bw of the clove oil nanoemulsion, and IM or CPF at high doses in the drinking water. In male rats, IM and CPF caused a reduction in body weight gain and hepatotoxic effects as evidenced by increases in the liver enzymes AST, ALT, and ALP. They caused oxidative damage in the liver of male rats as indicated by the decreased liver activity of the GST, GPX, SOD, and CAT enzymes and decreased serum TAC. IM and CPF produced a significant dose-dependent increase in DNA damage in hepatocyte cells, resulting in moderate to severe liver damage with cells that are more inflammatory and have enlarged sinusoids and compacted nuclei. IM had a higher BMD than CPF for both body and liver weight, suggesting that CPF was more dose-dependently toxic than IM. Albumin was a highly sensitive liver biomarker for IM, while total protein was a biomarker for the CPF-treated rats. GPx was an extremely sensitive biomarker of oxidative stress in the IM treatment, while CAT and GPx were highly sensitive parameters in the CPF-treated rats. Therefore, at comparable doses, CPF has a higher potential to cause liver damage and oxidative stress than IM. The hepatotoxicity of IM and CPF can be mitigated by administering a nanoemulsion containing clove oil and pomegranate extract. The nanoemulsion acts as a protector against the oxidative stress caused by these insecticides, especially at high doses. The nanoemulsion based on clove oil increases the bioavailability and stability of the pomegranate extract, which has antioxidant properties.

## 1. Introduction

In both developed and developing countries, consumers as well as workers in the agricultural and public health sectors are regularly exposed to a variety of synthetic pesticides and their residues through occupational exposure or as a result of residues in food, water, the air, and the environment [1,2,3,4]. Commercial pesticide products contain active ingredient(s) in addition to a variety of inert substances that are used in the manufacturing of the formulation, including organic solvents, wetting agents, emulsifiers, stabilizers, and others. In reality, different pesticide formulations are used to control one or more pests together or sequentially according to the stage of the crop season. One of the challenges in toxicological studies is to evaluate the impact of concurrent exposure to multiple chemical compounds on human health. This strategy requires a more in-depth and integrated framework that can account for the interactions and synergy between various chemicals, compared to the standard procedure of evaluating the risk of chemicals individually. It is also essential to identify specific biomarkers that can reflect the combined effects of chemical mixtures on different biological pathways and systems. However, these complicated exposures to different chemicals may have cumulative toxic effects that are generally unexpected [1,2]. Thus, to evaluate the possibility of adverse health effects from pesticide exposure, toxicological information on active chemicals or formulations alone is not sufficient. In addition, to evaluate the possibility of cumulative adverse health effects from exposure to multiple pesticides, the current approach of assessing these chemicals one by one is not sufficient.

In Egypt, chlorpyrifos (an organophosphate insecticide) and imidacloprid (a neonicotinoid insecticide) are two commonly used insecticides for insect control in both the agriculture and public health sectors. These insecticides have been reported to alter oxidants/antioxidants status, cause biochemical and histopathological alterations, induce DNA and organ damage, and affect mammals’ health [5,6]. The toxicity of several pesticides is associated with the production of free radicals. These radicals are not only toxic themselves but are also involved in the pathophysiology of several diseases [7]. Furthermore, due to their involvement in the metabolic process that leads to the death of dopaminergic neuronal cells, they have been associated with pesticide-induced neurotoxicity [8]. The levels at which no observed adverse effects are observed for these insecticides are 14 mg/kg body/day weight for IM [9] and 1 mg/kg body weight/day for CPF [10].

Over recent decades, it has been widely accepted that plants have positive medical benefits [11,12]. The pharmaceutical industry uses around 20% of all known plants, which influences healthcare by supporting medical treatments against diseases such as cancer [13,14]. Plants can produce a broad spectrum of bioactive components that act as antioxidants and can protect against free radical damage and pesticide-induced oxidative damage [13,15]. In contrast to that of synthetic compounds, there has recently been an increase in interest in natural plant metabolite compounds for industrial and pharmacological usage [16,17]. A major limitation of using of EOs and plant extracts for human health is the lack of sufficient toxicity data for most of them. EOs and plant extracts are complex mixtures of various compounds that may have different effects on the human body. Therefore, it is essential to conduct rigorous toxicological studies for EOs and plant extracts before they can be safely used for human health purposes. These studies should follow the same standards and protocols as those for other chemicals, including dose–response assessments, acute and chronic toxicity testing, and evaluations of potential adverse effects.

The peel of the pomegranate (*Punica granatum* L.) plant, which possesses an antioxidant capacity, is a significant source of several bioactive metabolites [18,19]. It has demonstrated strong therapeutic advantages, including antioxidant and anticancer effects [20,21]. Essential oils (EOs) have been demonstrated to be highly effective and safe as anti-inflammatory, antioxidant, anti-diabetic, cancer-preventive, hyperpigmentation-reducing, calming, antibacterial, antiviral, and antifungal compounds [22,23]. Clove (*Syzygium aromaticum* L.) essential oil is one of the most effective antioxidants and free radical scavengers. The high levels of polyphenolic chemicals in clove oil have the potential to improve liver health [24,25]. Cinnamon EO has strong biological effects against bacteria and fungi, as well as antioxidant and antidiabetic effects. It has been applied as a nematicidal, insecticidal, anticancer, and anti-inflammatory drug. This activity is due to the presence of trans-cinnamaldehyde, eugenol, linalool, and other bioactive components [26,27,28,29,30]. Regarding the safety of clove oil and its possible effects on human health, the Canadian government has carefully evaluated clove oil and its main component, eugenol, based on recent studies. The findings of their assessment show that clove oil and eugenol are not detrimental to human health when taken in acceptable dosages [31]. The no-observed-adverse-effect level (NOAEL) for pomegranate fruit extract, which corresponds to the maximum dose evaluated in the study by Chintan et al. [32], was established as 600 mg/kg body weight/day.

In fact, poor water solubility and stability, limited bioavailability, and significant changes because of their first-pass metabolism limit the therapeutic potential of EOs and plant-based bioactive compounds. To increase the bioavailability and absorption of bioactive components, nanoemulsions may be helpful. Previous studies have shown that using nanoformulations improves the delivery of bioactive ingredients, enhancing a number of pharmacokinetic properties and increasing the therapeutic value of phytochemicals [33,34]. Hence, it has been established that nano phytoconstituents are more effective than native phytoconstituents in terms of their bioavailability and therapeutic efficacy [35,36]. It may be beneficial to use nanoemulsions to increase the bioavailability and absorption of beneficial compounds [37,38].

The benchmark dose (BMD) was designed as an alternative for risk evaluations due to the limitations of the no-observed-adverse-effect level (NOAEL) [39,40]. A BMD is a statistically lower confidence limit for a dosage causing a predefined increase in the response rate, such as 0.01 or 0.1. To calculate the BMD, a mathematical dose–response model is used. This method effectively uses the sample size and dose–response curve form [39,41]. The sex of the animals used in toxicological studies is one of the crucial criteria that should be taken into consideration. The toxicokinetics and toxicodynamics of xenobiotics can be affected by sex variations in behavior, exposure, anatomy, physiology, biochemistry, and genetics, resulting in differing reactions and effects in males and females. For example, sex hormones can influence the distribution, metabolism, excretion, and absorption of xenobiotics. Consequently, we used male rats as animal models for this study. To establish accurate comparisons, it is crucial to include both sexes in toxicity investigations [42].

The aim of the current work is to evaluate the adverse toxic effects of the insecticides imidacloprid and chlorpyrifos on DNA damage, oxidative stress, liver function, and damage in male rats. Furthermore, the novel pomegranate-peel-extract-loaded nanoemulsions are evaluated for their possible protective properties to reduce oxidative stress, DNA damage, and hepatotoxicity against IM and CPF. The most sensitive biomarkers are identified using the BMD technique, which was also used to assess the dose–response toxicity of chlorpyrifos and imidacloprid.

## 2. Materials and Methods

### 2.1. Insecticides

The neonicotinoid insecticide Imidor 35% SC, commonly known as imidacloprid (IM), was purchased from ASTRCHEM, kingdom of Saudi Arabia Dammam—Second Industrial Area. Its chemical name is 1-[(6-chloro-3-pyridinyl) methyl]-N-nitro-2-imidazolidinimine. Chlorfan 48% EC, commonly known as chlorpyrifos (CPF), is an organophosphate insecticide that was purchased from Kafr El-Zayat Pesticides and Chemicals Company, Egypt. It is chemical name is O, O-diethyl O-(3,5,6-trichloro-2-pyridinyl) phosphorothioate.

### 2.2. Pomegranate-Peel-Extract-Loaded Clove Oil Nanoemulsion

In our previous study, clove oil was formulated in an oil-in-water (O/W) nanoemulsion at a concentration of 10% using deionized water and the nonionic surfactant polysorbate 80 (Tween 80 was purchased from VWR International, located at 201 Rue Carnot in Fontenay/Bois, France) (untabulated data). Clove oil nanoemulsion (sample B3) was prepared at 1:2 ratio of clove oil: Tween 80 with a sonication time of 10 min, using ultrasonic sonicator (Sonics & Materials, INC., 53 Church Hill Rd. Newtown, CT, USA) with a droplet size of 155.2 nm (using a dynamic light scattering instrument (DLS, PSS, Santa Barbara, CA, USA)). Then one gram of pomegranate extract was mixed with 35 mL of distilled water to create the aqueous phase, which was then used to prepare the clove nanoemulsion [43]. A pomegranate–clove nanoemulsion was then formed by sonicating the extract-loaded nanoemulsion for 10 min at room temperature. The final concentration of pomegranate extract was 20 mg/mL, which was used to calculate doses in experimental animal studies. The dose of the clove-oil-based nanoemulsion loaded with pomegranate extract was chosen to be 50 mg/kg body weight of the extract and 500 mg/kg body weight of clove oil and was administered orally (1 mL/100 g body weight of PELCN) according to the results of antioxidant activity studies and previous studies [44,45].

### 2.3. Animals and Experimental Design

For this study, 50 male Albino rats (*Rattus norvegicus*), each weighing 145–155 g at seven weeks of age, were used. The experiments were carried out at the National Research Center’s (NRC) Animal Breeding House (ABH), Dokki, Egypt. Before treatment, they had seven days to acclimatize. Five rats were housed in clean cages at ABH, where the animals were kept in a controlled environment with a temperature of 22 °C, light cycles of 12:12, good ventilation, a standard pellet diet, and water *ad libitum*. The National Research Centre, Cairo, Egypt’s Animal Care & Experimentation Committee (approved no. 12115062022) approved the experimental study on rats, and it was carried out in accordance with the guidelines for the ethical care and use of laboratory animals [46].

In this experiment, ten groups of five rats each (groups I, II, III, IV, V, VI, VII, VIII, IX, and X) were used. The animal groups and treatments are as follows:

- Group (I): control group, which received only drinking water.
- Group (II): group that received pomegranate-extract-loaded clove nanoemulsion (PELCN), which administered orally (1 mL/100 g/kg bw/day) at a dose equal to 50 mg/kg bw/day of pomegranate extract and 500 mg/kg bw/day of clove oil nanoemulsion.
- Groups (III, IV and V): imidacloprid (IM) groups, which received IM in drinking water at doses of 14, 28, and 54 mg/kg bw/day, respectively. The lowest dose level of IM that was selected was equal to the NOAEL (14 mg/kg bw/day), whereas the two- and four-fold doses were 28 and 54 mg/kg bw/day, respectively [9].
- Groups (VI, VII, and VIII): chlorpyrifos (CPF) groups, which received CPF in drinking water at doses of 1, 2, and 4 mg/kg bw/day, respectively. The lowest dose level of CPF that was selected was equal to the NOAEL (1 mg/kg bw/day), whereas the other doses were 2 and 4 times the NOAEL [10].
- Group (IX): IM-PELCN group, which received oral PELCN at a dose of 50 mg/kg bw/day of pomegranate extract and 500 mg/kg bw/day of clove oil nanoemulsion, as well as IM in drinking water at a dose of 54 mg/kg bw/day.
- Group (X): CPF-PELCN group, which received oral PELCN at a dose of 50 mg/kg bw/day of pomegranate extract and 500 mg/kg bw/day of clove oil nanoemulsion as well as CPF in drinking water at a dose of 4 mg/kg bw/day. All treatments were administered every day for 28 days. The IM and CPF dosages were chosen in accordance with Organisation for Economic Cooperation and Development (OECD)’s guidelines; the highest dose level produced toxic effects but not severe injury or death. The lowest dose was equal to the no-observed-adverse-effects level (NOAEL), whereas the highest dosages were equivalent to two and four times the NOAEL [47]. Animals were fasted overnight at the end of the study. Body weights were recoded daily, and based on the average daily water intake and body weights of the treated male rats, daily adjustments to the IM and CPF doses were made before being administered in drinking water. The dose of pomegranate-extract-loaded clove nanoemulsion was chosen at 50 mg/kg bw/day of extract and 500 mg/kg bw/day of clove oil and was administered orally (1 mL/100-g bw/day of PELCN) according to the results of antioxidant activity studies and previous studies [44,45].

### 2.4. Sample Collecting

Blood and organ samples were collected by skilled technicians at the ABH of the NRC, under anesthetization with pentobarbital at the lowest dose (40 mg/kg) intraperitoneally (IP). Using a sterilized fine glass capillary tube, blood samples were taken from the retro-orbital venous plexus and collected in two vacutainer tubes for serum and an EDTA tube for plasma. All blood samples were centrifuged at 2500 rpm for 10 min at 4 °C using a Heraeus Labofuge 400R (Kendro Laboratory Products GmbH in Germany) to collect the serum or plasma. Following that, serum or plasma samples were kept at −20 °C and utilized within a week for biochemical analysis. The liver samples were then taken from the rats, weighed, and divided into three portions: one for an oxidative stress study, one for a comet assay, and one for histological research. For oxidative stress and comet assay, the samples were stored in a freezer at −20 °C and used immediately. For the histological study, the samples were preserved in 10% formalin until further processing.

### 2.5. Biomarkers of Serum Liver Function

Serum liver function biomarkers, such as aspartate aminotransferase (AST; EC. 2.6.1.1), alanine transaminase (ALT; EC. 2.6.1.2) [48], alkaline phosphatase (ALP; E.C. 3. I. 3.1) [49], total bilirubin [50], total protein [51], TP, and albumin [52], were analyzed in accordance with the instructions provided by Biodiagnostic Kits, 29 Tahreer St., Dokki, Giza, Egypt.

### 2.6. Plasma Total Antioxidant Capacity (TAC)

According to Koracevic et al. [53] the total antioxidant capacity of plasma was assessed using the instructions provided by Biodiagnostic Kits, 29 Tahreer St., Dokki, Giza, Egypt.

### 2.7. Oxidative Stress Biomarkers in Liver Homogenate

#### 2.7.1. Liver Tissue Homogenate

The liver tissues were homogenized at a ratio of 1:10 in phosphate-buffered saline (100 mM, pH 7.4). The samples were then centrifuged for 15 min at 4 °C at 10,000 rpm. The obtained supernatant was used to determine the markers for oxidative stress.

#### 2.7.2. Antioxidant Enzymes in Liver Homogenate

Superoxide dismutase (SOD, EC 1.15.1.1) [54], was determined by the creation a red formazan dye when 2-(4-iodophenyl)-3-(4-nitrophenol)-5-phenyltetrazolium chloride (INT) interacts with superoxide radicals produced by the reaction of xanthine with xanthine oxidase. Glutathione-s-transferase (GST, EC 2.5.1.18) was measured based on the method of the conjugation of reduced glutathione and S-2,4-dinitrophenyl glutathione (CDNB). By comparing the net increase in absorbance at 340 nm to the blank, the development of the CDNB, S-2,4-dinitrophenyl glutathione adduct was measured [55]. Glutathione peroxidase (GPX, EC 1.11.1.9) was determined according to the method of Paglia and Valentine [56]. Catalase (CAT, EC 1.11.1.6) was determined according to the method reported by Aebi [57]. The method is dependent on catalase’s breakdown of H_2_O_2_. The reaction mixture included liver homogenate, 10 mM H_2_O_2_, and 50 mM phosphate buffer at a pH of 7.0.

### 2.8. Comet Assay (Single-Cell Gel Electrophoresis) of Liver Samples

According to OECD guidelines [58], a modified comet assay was used to evaluate how IM and CPF damaged DNA using hepatocytes by single-cell gel electrophoresis as cited in previous studies [40,59]. After the usual steps of washing, embedding the separated cells in agarose gel on microscope slides, lysing, and conducting the alkali treatment for 20 min, electrophoresis was performed (30 min, 300 mA, 25 V). Subsequently, following ethidium bromide staining, fluorescence microscopy (LEICA DM 2500 with filter N2.1) was used to check 100 cells for DNA damage (×40). DNA damage in the overlapping cells was assessed and graded as follows: class 0 for no detectable DNA damage and no tail; class 1 for a tail that is shorter than the nuclear diameter; class 2 for a tail that is longer than the nuclear diameter; and class 3 for a tail that is longer than 2 times the nuclear diameter.

### 2.9. Benchmark Dose (BMD)

Dose–response curves for all parameters were obtained using PROAST version 70.1 (https://proastweb.rivm.nl/, accessed on 31 August 2022). A benchmark dose (BMD) was calculated to determine the most sensitive biomarkers.

### 2.10. Histopathological Analysis Techniques

Dissected liver specimens were preserved in 10% formalin, dehydrated, cleared in xylene, and embedded in paraffin wax. The tissues were divided into 5 μm thick sections. Sections were then stained with hematoxylin and eosin. Two slides containing two sections each were prepared for each rat. Ten field areas were selected for each slice, and histopathological changes were checked by light microscopy (×160). According to Michael [60], the organ fields were graded as follows: normal (-), minimal cellular disruption in less than 1% of the field area (+), mild cellular disruption in between 1% and 30% of the field area (++), moderate cellular disruption in between 31% and 60% of the field area (+++), severe cellular disruption in between 61% and 90% of the field area (+++++), and very severe cellular disruption in between 91% and 100% of the field area (+++++).

### 2.11. Statistical Analyses

Data from this study were analyzed using SPSS software v26.0 (IBM Corp., Armonk, NY, USA) and expressed as mean ± standard deviation. Data were analyzed using one-way ANOVA followed by a post hoc test for the least significant difference. Statistical significance was set at *p* ≤ 0.05.

## 3. Results

### 3.1. Pomegranate-Peel-Extract-Loaded Clove Oil Nanoemulsion

The objective of this study was to investigate the solubility of pomegranate extract in clove oil and its nanoemulsion at various concentrations. A nanoemulsion of clove oil was prepared using a high-pressure homogenization method and then mixed with pomegranate extract to obtain a pomegranate–clove oil nanoemulsion. The concentration of pomegranate extract in the nanoemulsion was 25 mg/mL, which was determined based on the experimental design for animal studies.

### 3.2. Signs of Toxicity, Body and Liver Weights

The effects of IM and CPF on the body and liver weights of the rats were evaluated during the treatment period. Table 1 summarizes the results of these parameters. The rats exposed to IM and CPF showed no sign of toxicity. However, both the IM and CPF treatments reduced the body weight gain of the rats significantly (*p* ≤ 0.05) compared to the control group. The highest doses of IM (group V) and CPF (group VIII) resulted in the lowest body weight gains, which were 17.55 g (−10.91% of the control) and 16.45 g (−16.49% of the control), respectively, while the control group gained 19.70 g. On the other hand, the co-treatment with the pomegranate extract loaded with a clove-oil-based nanoemulsion (groups IX and X) ameliorated the body weight loss induced by IM or CPF and increased the body weight gains to 18.50 g (−6.09% of the control) and 17.95 g (−8.88% of the control), respectively. The relative liver weight was also affected by the IM and CPF treatments. The control group had a relative liver weight of 3.11%, while the IM and CPF groups had 3.39% and 3.59%, respectively (Table 1). This indicates an enlargement of the liver due to IM or CPF exposure. However, the co-treatment with the pomegranate extract loaded with a clove-oil-based nanoemulsion (groups IX and X) normalized the relative liver weight to 3.12% and 3.02%, respectively, suggesting a protective effect of the nanoemulsion on the liver.

### 3.3. Liver Function Markers

The effects of IM and CPF on the liver function of male rats were investigated. In this study, we evaluate the changes in liver function biomarkers, such as AST, ALT, ALP, total bilirubin, total protein, and albumin, in male rats treated with different doses of IM or CPF alone or in combination with a clove-oil-based nanoemulsion loaded with pomegranate extract. The results showed that the exposure of the male rats to different doses of IM (groups III, IV, and V) or CPF (groups VI, VII, and VIII) resulted in changes in the liver function biomarkers AST, ALT, ALP, total bilirubin, total protein, and albumin. These findings indicate that both IM and CPF have hepatotoxic effects on male rats, as evidenced by the significant increases in liver enzymes (Table 2). In particular, the total bilirubin and total protein levels were increased while the albumin levels were decreased in the treated rats compared to the control rats (Table 2). The results also showed that the liver function of the rats was improved by co-administering a nanoemulsion based on clove oil and containing pomegranate extract along with the IM or CPF treatment (groups IX and X). This is evidenced by the reduced levels of AST, ALT, and ALP, and the increased levels of total bilirubin, total protein, and albumin.

### 3.4. Oxidative Stress Markers

The results revealed that IM and CPF exposure significantly reduced the activity of GST, GPX, SOD, and CAT enzymes in the liver and decreased the serum total antioxidant capacity (TAC) in groups III to VIII (Table 3). These changes indicated that IM and CPF induce oxidative damage in the liver and impair its ability to cope with oxidative stress, which may increase the risk of liver disease. On the other hand, the administration of the nanoemulsion enhanced the antioxidant enzyme activity in the liver and increased the serum TAC. Moreover, the co-administration of the nanoemulsion with IM and CPF (groups IX and X) attenuated their hepatotoxic effects, especially at the highest doses. These findings suggested that the nanoemulsion has a beneficial effect against IM- and CPF-induced oxidative stress in the liver.

### 3.5. DNA Damage

**Figure 1:**
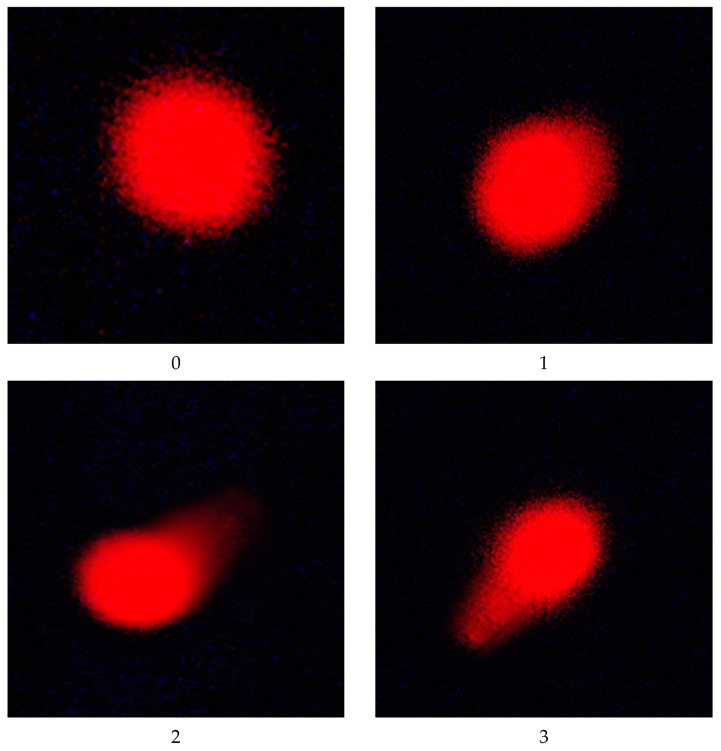
Normal (class 0) and DNA-damaged (classes 1, 2, 3) hepatocyte cells of male rats exposed to imidacloprid and chlorpyrifos at different doses for 28 days using comet assay.

Using a comet assay, we investigated the effects of IM and CPF on DNA damage in hepatocyte cells. We measured the percentage of cells with DNA damage and the tail length of the damaged DNA to assess the DNA damage. The results revealed that IM and CPF caused a significant dose-dependent increase in DNA damage in hepatocyte cells compared to that of the untreated control group (Table 4 and Figure 1). The percentage of cells with DNA damage rose from 9.75% in the control group to 23.75% and 24.25% in the groups treated with the highest doses of IM and CPF (groups V and VIII), respectively. In contrast, the groups receiving the pomegranate-extract-loaded clove-oil-based nanoemulsions showed a significant decrease in tail length to 15.75 and 16.50 (groups IX and X) in comparison with IM and CPF, respectively.

### 3.6. Histopathological Study

**Figure 2:**
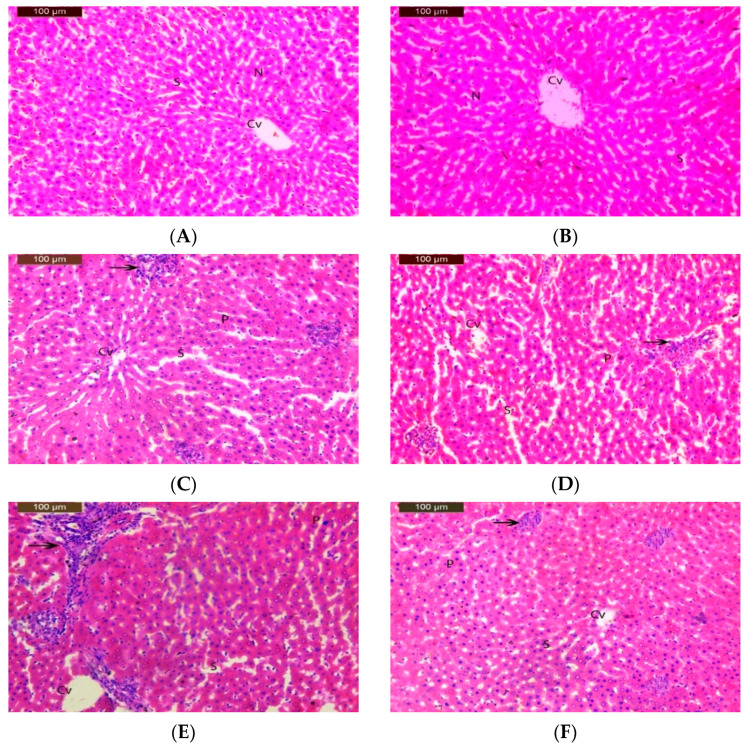

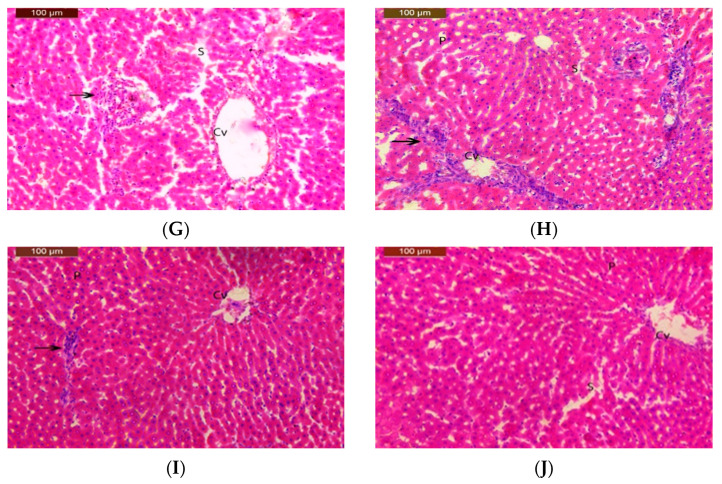
Photomicrography of liver sections showing normal liver tissue in (**A**) control group and (**B**) the 50 mg/Kg nano-emulsion treatment group with normal central vein (cv), blood sinusoids (S), and nucleus (N). (**C**,**D**) Imidacloprid-treated groups at conc. of 14 and 28 mg/kg bw/day. (**F**,**G**) Chlorpyrifos-treated groups at conc. of 1 and 2 mg/kg bw/day, showing few inflammatory cell infiltrations (arrow), dilated sinusoids (S), and pyknotic nuclei (P). (**E**) Imidacloprid-treated group at conc. of 54 mg/kg bw/day. (**H**) Chlorpyrifos-treated group at conc. 4 of mg/kg bw/day showing moderate degeneration changes with increased inflammatory cell infiltration (arrow), dilated sinusoids (S), and pyknotic nuclei (P). (**I**) Imidacloprid group at conc. of 54 mg/kg bw/day plus 50 mg/kg bw/day nano-emulsion treatment. (**J**) Chlorpyrifos group at conc. of 4 mg/kg bw/day plus 50 mg/kg bw/day nano-emulsion showing almost normal structure with few dilated sinusoids (S) and pyknotic nuclei (P). (H&E X 200).

A liver histology of the rats exposed to clove-oil-based nanoemulsion loaded with pomegranate extract, IM, and CPF is shown in Table 5 and Figure 2. The control rats showed a normal liver architecture with a central vein, hepatocytes, hepatic sinusoids, and clear nuclei. The rats receiving low and medium doses of IM (groups III and IV) or CPF (groups VI and VII) showed mild liver damage with some inflammatory cells, enlarged sinusoids, and condensed nuclei. The high doses of IM (group V) or CPF (group VIII) caused moderate-to-severe liver damage with more inflammatory cells, enlarged sinusoids, and condensed nuclei. The rats receiving IM or CPF along with a clove-oil-based nanoemulsion loaded with pomegranate extract (groups IX and X) showed an improved liver architecture with fewer inflammatory cells, enlarged sinusoids, and condensed nuclei. The liver histology of both the control (group I) and the pomegranate-extract-loaded clove oil nanoemulsion treatment group (group II) showed a typical structure with no signs of damage. However, the high-dose treatment with IM and CPF caused moderate damage to the liver tissue, as indicated by the score ++. The co-administration of the pomegranate-loaded clove oil nanoemulsion reduced the severity of liver damage to a mild level (+), as shown in Table 5.

### 3.7. Benchmark Dose (BMD) Approach

**Figure 3:**
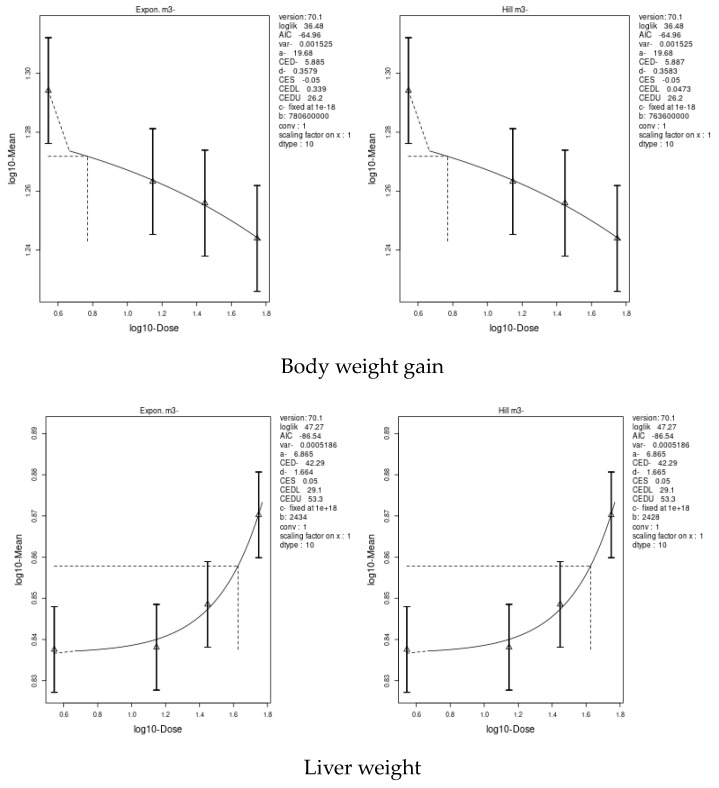
Dose–response curve for body and liver weights in male rats exposed to imidacloprid (IM) at different doses for twenty-eight consecutive days. Curves were obtained using PROAST version 70.1.

**Figure 4:**
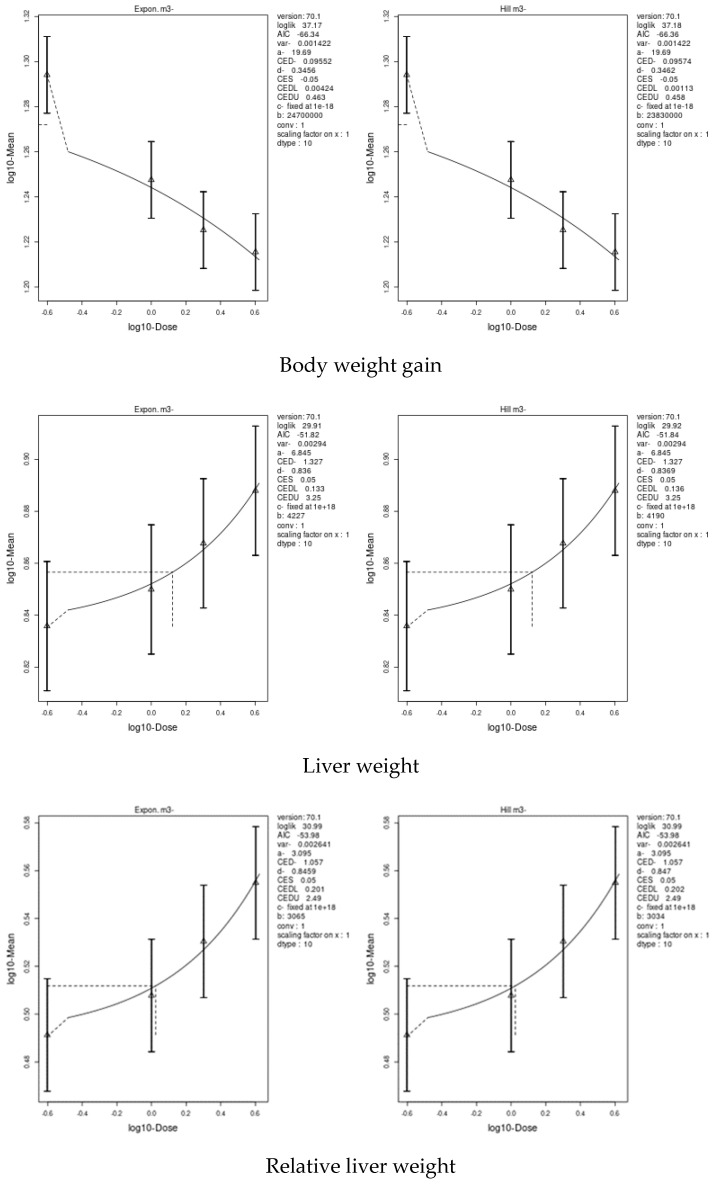
Dose–response curve for body, liver, and relative liver weights in male rats exposed to chlorpyrifos (CPF) at different doses for twenty-eight consecutive days. Curves were obtained using PROAST version 70.1.

**Figure 5:**
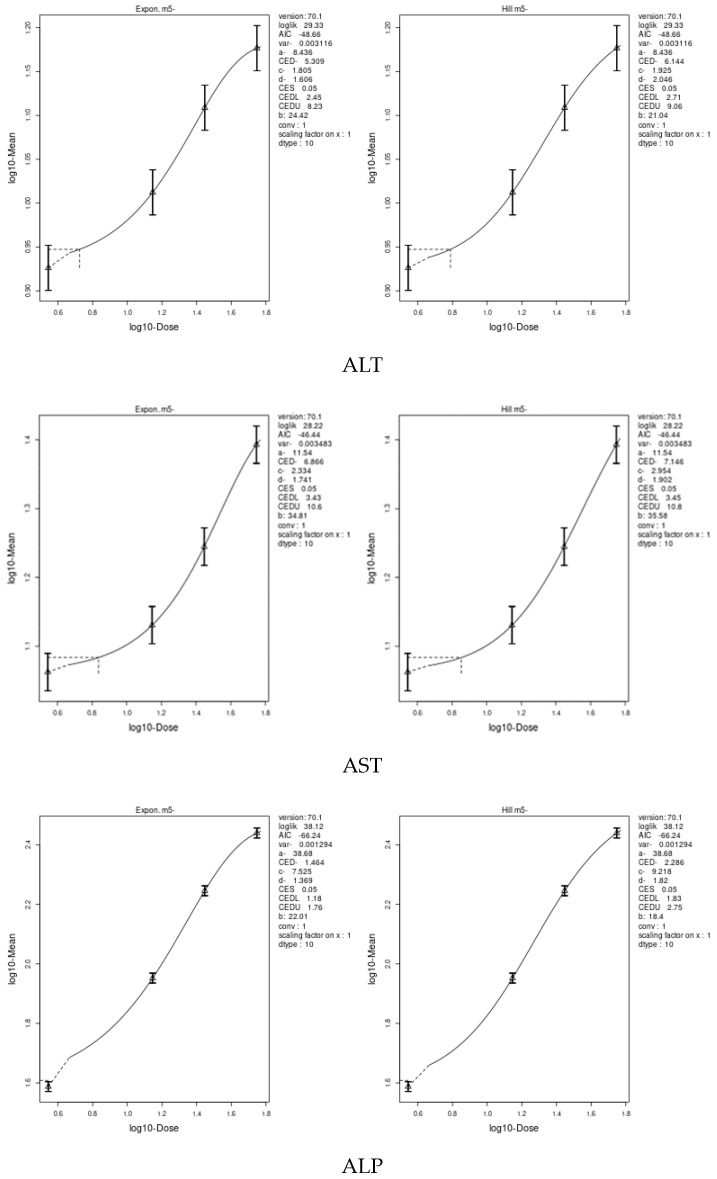

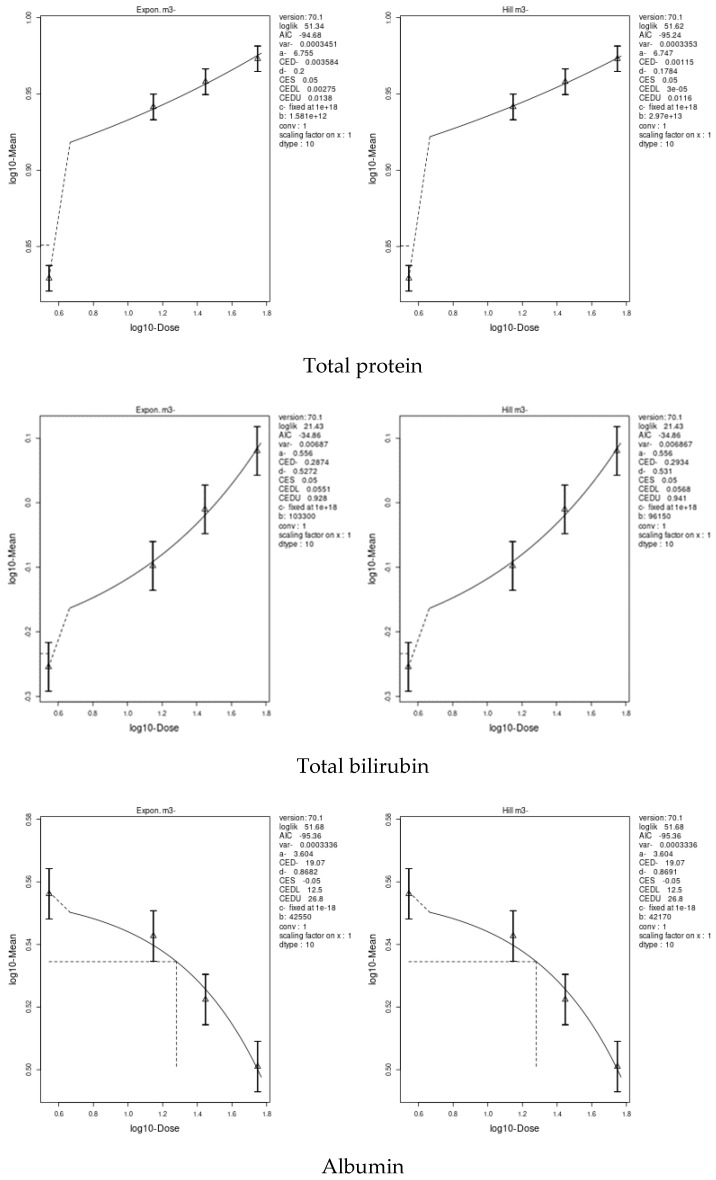
Dose–response curve for liver markers (ALT, AST ALP, total protein, total bilirubin, and albumin) in male rats exposed to imidacloprid (IM) at different doses for twenty-eight consecutive days. Curves were obtained using PROAST version 70.1.

**Figure 6:**
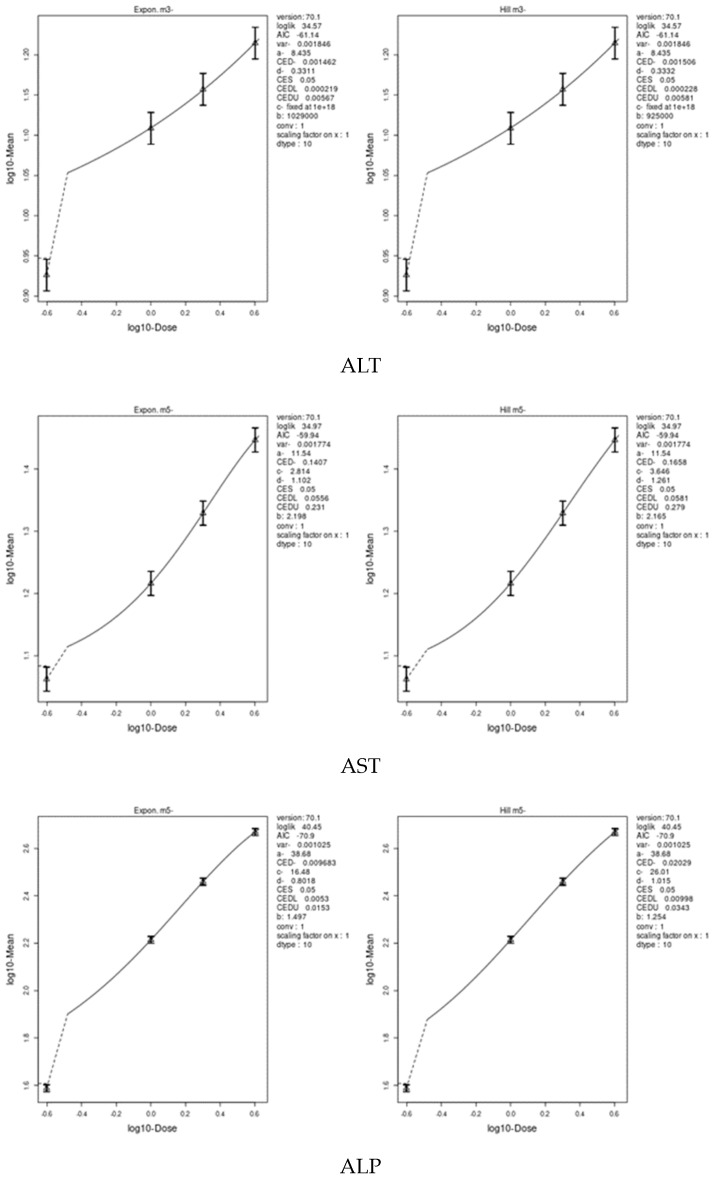

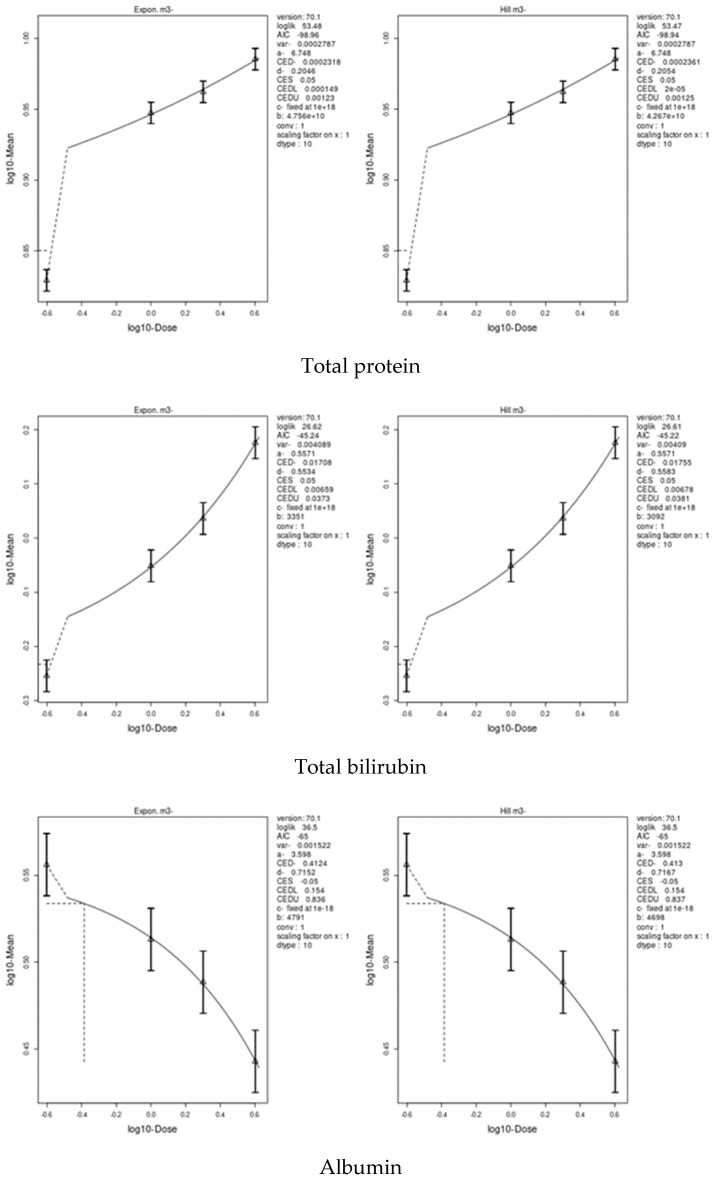
Dose–response curve for liver markers (ALT, AST ALP, total protein, total bilirubin, and albumin) in male rats exposed to chlorpyrifos (CPF) at different doses for twenty-eight consecutive days. Curves were obtained using PROAST version 70.1.

**Figure 7:**
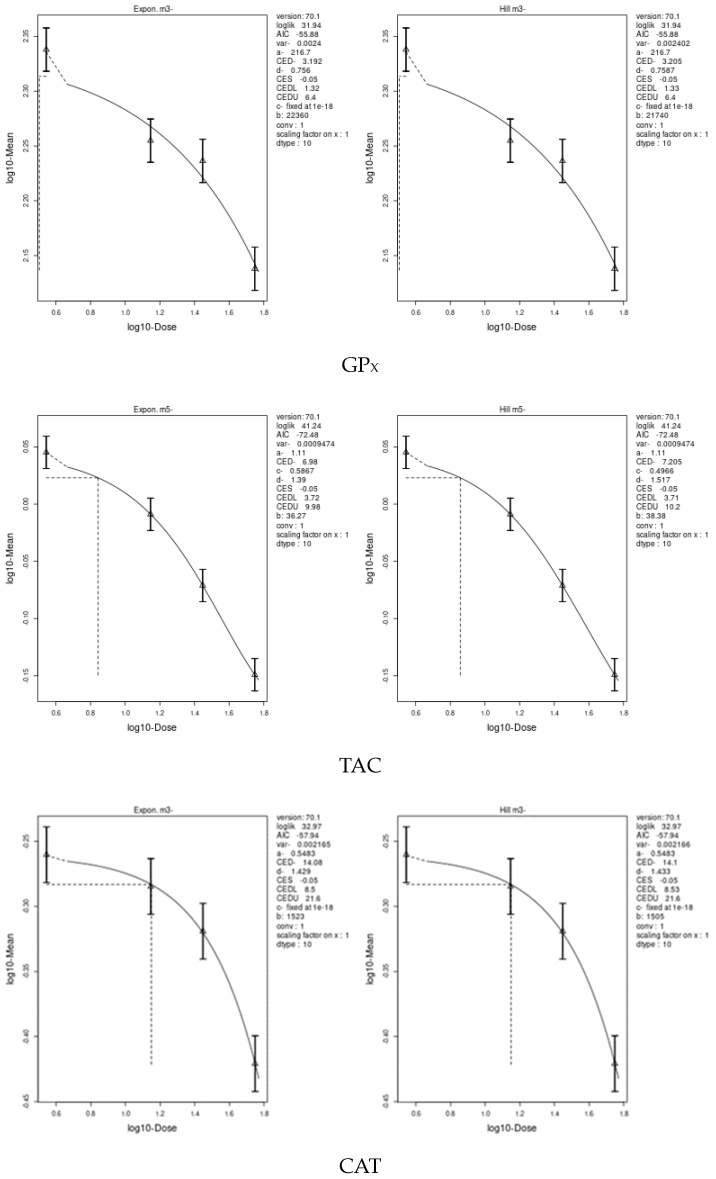

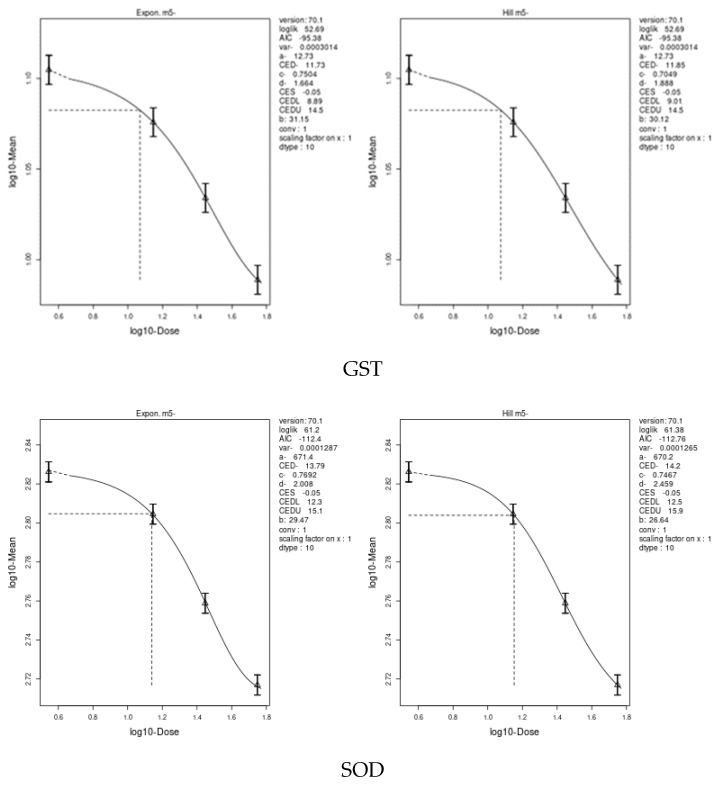
Dose–response curve for oxidative stress markers (GP_X_, TAC, CAT, GST, and SOD) in male rats exposed to imidacloprid (IM) at different doses for twenty-eight consecutive days. Curves were obtained using PROAST version 70.1.

**Figure 8:**
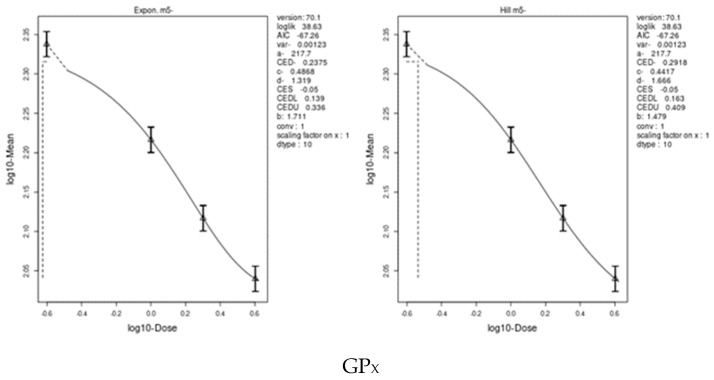

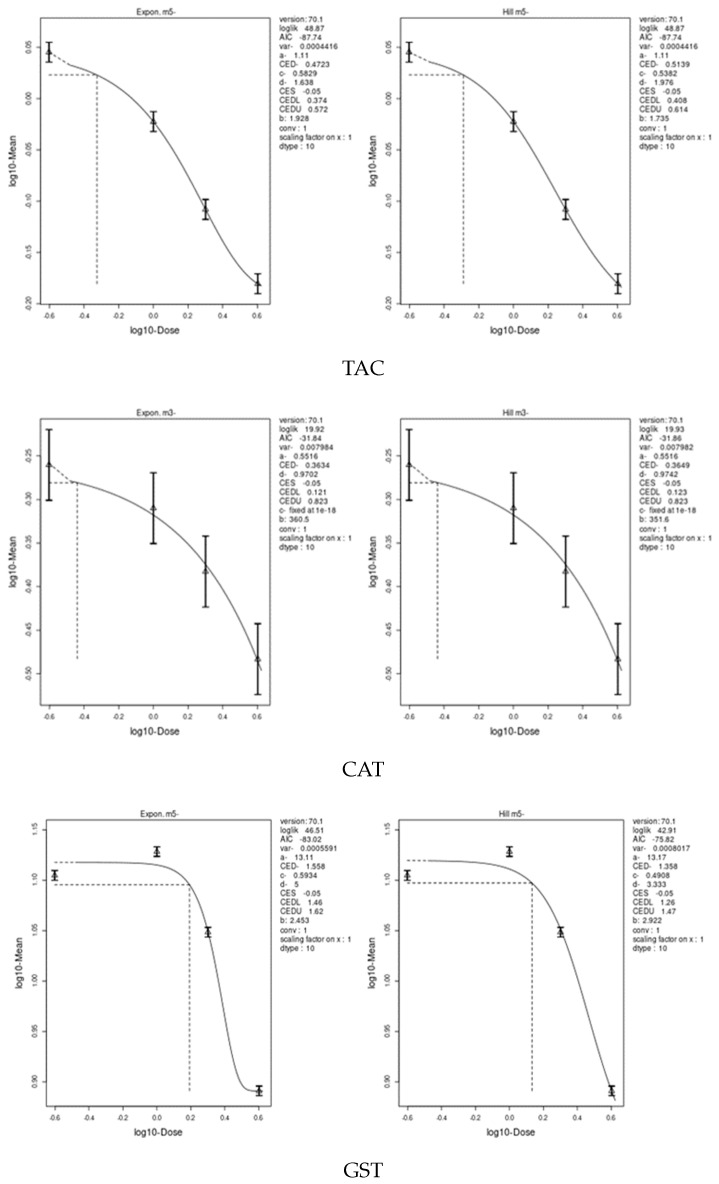

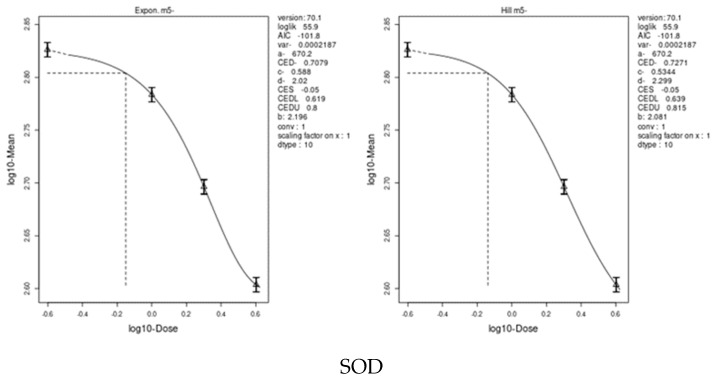
Dose–response curve for oxidative stress markers (CAT, GP_X_, TAC, SOD, and GST) in male rats exposed to chlorpyrifos (CPF) at different doses for twenty-eight consecutive days. Curves were obtained using PROAST version 70.1.

We applied two models, an exponential model and the Hill model, to fit the dose–response curves of the body and liver weight for IM and CPF. We calculated the benchmark doses (BMDs) for each parameter using these models. The BMDs of the body and liver weight for IM were 5.885 and 42.29 mg/kg bw/day, respectively. The BMDs of the body, liver, and relative liver weight for CPF were 0.09552, 1.327, and 1.057 mg/kg bw/day, respectively (Table 6 and Figure 3 and Figure 4). We also estimated the BMD of the liver markers and oxidative stress markers for both insecticides. The BMD of the liver markers for IM ranged from 0.00115 to 19.07 mg/kg bw/day, while the BMD of the liver markers for CPF ranged from 0.0002361 to 0.4124 mg/kg bw/day (Table 6 and Figure 5 and Figure 6). The BMD of the oxidative stress markers for IM ranged from 3.192 to 14.08 mg/kg bw/day, while the BMD of the oxidative stress markers for CPF ranged from 0.2375 to 1.358 mg/kg bw/day (Table 6 and Figure 7 and Figure 8).

## 4. Discussion

### 4.1. Adverse Effects of Exposure to Pesticides

Pesticides have important impacts on agriculture and human health with respect to vector-borne disease prevention and crop pest control. They also improve food production. Despite their importance, pesticides have negative effects, such as toxic residues in food, water, the air, and soil, and effects on organisms other than their intended targets, such as humans, animals, birds, and aquatic organisms [61,62]. The World Health Organization (WHO) and United Nations Environment Programme (UNEP) report that pesticides pose a major threat to human health through direct or indirect exposure, with more than 26 million people suffering from pesticide poisoning and nearly 220,000 deaths each year [63]. In addition to the common toxic mechanism, neonicotinoids and organophosphate pesticides promote free radical production and can alter the oxidant–antioxidant balance when exposed to very low levels [8,40,64]. The human body has various defense mechanisms to protect against free radical species (FRS)/reactive oxygen species (ROS) damage, including enzymatic and nonenzymatic pathways [65,66].

Farmers are one of the most vulnerable groups as they are exposed to higher levels of pesticides than consumers are. These pesticides have significant health effects, particularly for farm workers in poor countries, who have been exposed to pesticides primarily during the preparation and application of spray solutions [67,68]. At low doses, the toxicity of pesticides is mainly related to their production of free radicals. The continuous accumulation of these radicals with chronic and sub-chronic exposure to pesticides has various adverse biochemical, genetic, and pathophysiological effects, which are the main factors in various diseases [69,70].

### 4.2. Clove-Oil-Based Nanoemulsion Loaded with Pomegranate Extract

Using a self-emulsification method with Tween 80 as a surfactant, we developed a nanoemulsion of clove oil in our previous study. We adjusted the oil-to-surfactant ratio and the sonication time to achieve the optimal nanoemulsion with a droplet size of 155.2 nm and desirable physicochemical properties. In the current study, different concentrations of pomegranate extract were mixed with clove oil or its nanoemulsion. The amount of extract in the nanoemulsion (25 mg/mL) was calculated to set doses for experimental animal studies. However, many plants have natural antioxidants that scavenge free radicals and protect humans from mutagenesis, carcinogenesis, and aging. Bioactive antioxidant compounds, such as the phenolic content of plants, are of great importance because the hydroxyl group confers antioxidant activity, making them versatile natural molecules with promising therapeutic and protective applications [71]. The current study evaluated CPF and IM toxicity in male rats and examined the in vitro antioxidant, anti-DNA-damage, and hepatoprotective activities of essential oil nanoemulsions containing plant extracts.

### 4.3. Effect of IM and CPF on Body and Liver Weights

IM and CPF showed no signs of toxicity over the course of treatment. Both the IM and CPF treatments caused a decrease in body weight gain especially at high doses, which induced significant (at *p* ≤ 0.05) decreases. In contrast to IM or CPF treatments, the co-administration of pomegranate extract loaded with a clove-oil-based nanoemulsion increased body weight gain in the treated rats. Important endpoints for assessing the toxicity of pesticides are toxicological symptoms and body and organ weights [5,72,73,74]. Other studies have found a reduction in body weight gain after exposure to pesticides such as CPF [75,76] and IM [77,78]. This reduction in body weight gain may be caused by the interaction of cholinergic and oxidative stress [79], as well as increased protein and lipid breakdown which is a direct result of exposure to insecticides [80,81].

In the current study, the IM and CPF treatments increased the relative liver weight of the treated rats. The ratio of a rat’s liver weight to its body weight is referred to as its relative liver weight. Toxicology studies often use it to determine how a treatment may affect the liver [82,83]. Previous studies in rats have shown that chlorpyrifos [84,85] and imidacloprid [86] can cause an increase in liver weight. This increase in liver weight could be due to the hepatotoxicity of chlorpyrifos and imidacloprid. According to other studies, increased liver and organ weights are closely correlated with hepatocellular hypertrophy [87,88]. The co-administration of pomegranate extract loaded with a clove-oil-based nanoemulsion improved the liver and relative liver weights of the rats treated with IM or CPF.

### 4.4. Effect of IM and CPF on Liver Function Biomarkers

The liver is an important organ that plays a crucial role in detoxification and the metabolism of pesticides [5,85]. Pesticides can cause liver damage and induce liver dysfunction, including changes in ALT, AST, ALP, serum bilirubin, total protein, and albumin, which are commonly used as liver function tests [85,89,90]. The exposure of the male rats to different doses of IM or CPF resulted in changes in the liver function biomarkers AST, ALT, ALP, total bilirubin, total protein, and albumin. These findings indicated that both IM and CPF have hepatotoxic effects on male rats as evidenced by the significant increases in AST, ALT, and ALP liver enzymes. AST, ALT, and ALP are liver enzymes mainly produced by liver cells. These enzymes can enter the bloodstream and increase when the liver is damaged or inflamed. In particular, the total bilirubin and total protein levels were increased while the albumin levels were decreased in the treated rats compared to the control rats. One of the most common signs of liver damage is also changes in biochemical parameters in the serum, such as an increase in the total bilirubin and total protein and a decrease in albumin. These changes reflect the liver’s impaired function in metabolizing and excreting bilirubin and synthesizing proteins [91,92]. These findings suggest that IM and CPF have hepatotoxic effects on male rats. Our results also showed that the co-administration of a clove-oil-based nanoemulsion loaded with pomegranate extract with the IM or CPF treatment improved liver function. These results suggested that the use of a clove-oil-based nanoemulsion loaded with pomegranate extract could be a potential therapeutic approach to protect liver function from insecticide-induced toxicity. Further research is needed to determine the long-term safety and effectiveness of this therapeutic strategy. Other studies have reported on the hepatotoxicity of the widely used insecticides IM [93] and CPF [81,94,95]. Insecticides can disrupt the normal detoxification and metabolic functions of the liver, leading to oxidative stress, inflammation, fibrosis, and cell death [96,97]. CPF and IM are commonly used insecticides in agriculture, and their residues can be found in food and water sources. Long-term exposure to these chemicals can lead to chronic liver damage, which can have serious health consequences.

### 4.5. Effect of IM and CPF on Oxidative Stress Biomarkers

One of the major health risks associated with exposure to pesticides is oxidative stress, which occurs when the balance between reactive oxygen species (ROS) and antioxidants is disrupted [94]. ROS are molecules that can oxidize and damage various biomolecules, such as DNA, lipids, and proteins, leading to cellular disorders and diseases [98,99]. Pesticides can induce oxidative stress by increasing the production of ROS or decreasing the availability of antioxidants in biological systems. Therefore, it is important to understand the mechanisms of pesticide-induced oxidative stress and to develop strategies to prevent or mitigate its harmful effects. The results of this study showed that exposure to IM or CPF insecticides caused significant oxidative damage in the liver of male rats as indicated by decreased activity of the glutathione-S-transferase (GST), glutathione peroxidase (GPX), superoxide dismutase (SOD), and catalase (CAT) enzymes in the liver and the reduced serum antioxidant capacity (TAC). To protect itself from ROS, the liver relies on a system of antioxidant enzymes, such as GST, GPX, SOD, and CAT. These enzymes work together and reinforce each other to neutralize ROS and maintain liver redox homeostasis. GST is responsible for binding glutathione (GSH), a powerful antioxidant, to harmful electrophiles and facilitating their elimination. GPX uses GSH to reduce peroxides, such as H_2_O_2_ and organic peroxides, to harmless water and alcohol. SOD converts O_2_^-^, a highly reactive form of oxygen, into H_2_O_2_ and oxygen, preventing oxidative damage to cellular components. CAT breaks down H_2_O_2_ into water and oxygen and thus avoids its accumulation [98,100,101]. The reduced serum TAC is a measure of the blood plasma’s ability to scavenge free radicals and prevent oxidative damage. TAC reflects the combined action of various antioxidants, such as enzymes, vitamins, minerals, and proteins, present in plasma [102,103]. Therefore, TAC can be used as a biomarker of oxidative stress and antioxidant status in humans. Alterations in these enzymes can impair the liver’s ability to deal with oxidative stress and increase one’s susceptibility to liver disease. The administration of a clove-oil-based nanoemulsion loaded with pomegranate extract resulted in a significant increase in antioxidant enzyme activity. Combining the nanoemulsion with the IM and CPF treatments further reduced its hepatotoxicity, particularly at the highest doses. This indicates that the nanoemulsion has a protective function against IM- and CPF-induced oxidative stress.

### 4.6. Effect of IM and CPF on DNA Damage via Comet Assay

One of the methods to evaluate the genotoxic potential of pesticides is the use of single-cell gel electrophoresis (SCGE), also known as the comet assay [104,105]. This technique enables the detection of DNA strand breaks and alkali-labile sites in individual cells, which are indicators of DNA damage. The results from SCGE studies have shown that exposure to pesticides can increase the level of DNA damage in various cell types, which can lead to mutations, chromosomal aberrations, cancer, and other diseases [106,107]. Therefore, SCGE is a useful tool to assess the effect of pesticides on DNA damage and to monitor the genetic risk of exposed populations. The effects of IM and CPF on DNA damage in hepatocyte cells were studied using the comet assay. The DNA damage was assessed by measuring the percentage of cells with DNA damage and the tail length of the damaged DNA. The results showed that both IM and CPF induced a significant dose-dependent increase in DNA damage in hepatocyte cells compared to that of the untreated control group. These findings indicate that IM and CPF have genotoxic and cytotoxic potential in hepatocyte cells. On the other hand, the groups receiving the pomegranate-extract-loaded clove-oil-based nanoemulsion showed a significant reduction in tail length. This indicates that the nanoemulsion had a protective effect against liver DNA damage induced by IM and CPF. Other studies have reported that pesticides can induce DNA damage and the genotoxic use of single-cell gel electrophoresis (SCGE) [90,108]. SCGE can provide information about the type and extent of DNA damage, as well as the repair capacity of cells.

### 4.7. Effect of IM and CPF on Liver Tissue Architecture

Histopathological studies in experimental animals are essential to assess the effects of pesticides on organ function and structure. Depending on their dose, duration, and mode of action, pesticides can cause various types of liver damage, such as inflammation, necrosis, fibrosis, and tumors. Histopathological studies in laboratory animals can reveal the morphological and cellular changes in liver tissue and the possible mechanisms of pesticide-induced hepatotoxicity [40,109]. In the current study, the control rats showed a normal liver architecture while the livers of the rats receiving low and medium doses of IM or CPF showed mild liver damage with some inflammatory cells, enlarged sinusoids, and condensed nuclei. The high doses of IM or CPF caused moderate-to-severe liver damage with more cells that are inflammatory, enlarged sinusoids, and condensed nuclei. The rats receiving IM or CPF along with a clove-oil-based nanoemulsion loaded with pomegranate extract showed an improved liver architecture with fewer inflammatory cells, enlarged sinusoids, and condensed nuclei. The results of this study agree with those of Ojha et al. [110], who showed how organophosphorus insecticides damaged the liver of rats. In pesticide-treated rat livers, they saw central venous congestion and dilation, hepatocyte depletion, inflammatory cell infiltration, Kupffer cell growth, and fibrosis. The authors of [5,111,112] all observed similar histotoxic effects of CPF in rat livers. In rat livers treated with IM at higher doses, there was marked dilatation and congestion of the central vein and hepatocyte depletion. Other previous research supports the histological results of the current study in rats exposed to IM [113,114].

### 4.8. Benchmark Dose (BMD) Approach and Dose–Response

One of the challenges in risk assessment is determining a reference point (RP) or a reference dose (RfD). An RfD is a dose or concentration of a substance associated with a specified level of adverse health effects [115,116,117]. Two statistical approaches are currently available for identifying a reference point (RP): the no-observed-adverse-effect level (NOAEL) approach and the benchmark dose (BMD) approach [116,118]. The NOAEL approach is based on identifying the highest dose or concentration of a substance that causes no observable adverse effects in test animals or humans [119]. The BMD approach is based on fitting a mathematical model to the dose–response data and estimating the dose or concentration that corresponds to a predetermined level of effect, such as a 10% increase in the incidence of a specific effect [120].

The current study used two models, an exponential model and the Hill model, to determine the body and liver weight, liver, and oxidative stress biomarkers’ dose–response curves and to estimate the benchmark doses (BMD) for IM and CPF. The results showed that IM had a higher BMD than that of CPF for both the body and liver weight, indicating that CPF was more dose-dependently toxic than IM. Our study also found that body weight gain was the most sensitive biomarker for the IM and CPF treatments, as it had the lowest BMD scores among all parameters. The study concluded that IM and CPF have differential toxic effects on the body and liver weights in male rats and that body weight gain could be used as a reliable indicator of the toxicity of these insecticides. The BMDs were calculated for liver markers, such as the ALP, ALT, AST, total protein, albumin, and total bilirubin, which reflect liver function and damage. We also calculated the BMDs of oxidative stress markers, such as GPX, TAC, CAT, GST, and SOD, which indicate antioxidant status and free radical scavenging activity. Albumin was a highly sensitive liver biomarker for the IM-treated rats, while total protein was a biomarker for the CPF-treated rats. GPx was an extremely sensitive biomarker of oxidative stress in the IM treatment, while CAT and GPx were highly sensitive parameters in the CPF-treated rats. These results indicate that CPF has a higher potential to cause liver damage and oxidative stress than IM at comparable doses. Interestingly, the lower confidence limit of the BMD (BMDL) values for most of the body and organ weights, liver, and oxidative stress parameters in the current study were lower than the no-observed-adverse-effect level (NOAEL) values of IM and CPF reported in previous studies.

### 4.9. Mechanism of Hepatoprotective of Clove-Oil-Based Nanoemulsion Loaded with Pomegranate Extract

Our results showed the dose-dependent effects of IM and CPF on the liver, oxidative stress, DNA, and histopathological biomarkers in male rats. In fact, CPF itself has low persistence in the body, meaning it is rapidly metabolized (it has a half-life of 27 h in humans) and excreted. However, its active metabolites 3,5,6-trichloro-2-pyridinol (TCP) and chlorpyrifos-oxone (CPO) are more persistent and can accumulate in tissues and fluids. These metabolites are highly toxic [121,122]. IM can produce various toxic effects, and at lower doses, it can impair liver function, resulting in hepatotoxicity and jaundice. The main mechanism of toxicity of IM in humans is the inhibition of nAChRs involved in neuromuscular transmission and cognitive functions [123]. The hepatotoxicity of IM and CPF can be mitigated by administering a nanoemulsion containing clove oil and pomegranate extract. The nanoemulsion acts as a protector against the oxidative stress caused by these insecticides, especially at high doses. The clove-oil-based nanoemulsion increases the bioavailability and stability of pomegranate extract, which has antioxidant and anti-inflammatory properties [124,125]. A possible explanation for the beneficial effects of pomegranate extract and clove oil on the liver and oxidative stress is that they contain a large amount of phenolic and flavonoid compounds [126,127]. These compounds are known to have antioxidant properties and can protect liver cells from damage caused by free radicals and toxins. For example, eugenol is an important phenolic component of clove essential oil [128]. It has a high antioxidant potential and can modulate various biological processes due to its antioxidant properties [129]. In addition to its nutritional value, pomegranate extract is rich in phenolic compounds that have a beneficial effect on the liver. Some of these compounds are gallic acid, catechin, and ellagic acid, which have been shown to possess antioxidant and hepatoprotective properties [130]. However, one of the main functions of the liver is to detoxify the blood and remove toxins from the body. This process can also generate ROS, which are unstable molecules that can damage liver cells and cause oxidative stress [131,132]. Phenolic and flavonoid compounds can act as antioxidants by scavenging these ROS and preventing their accumulation in the liver [133]. They can also modulate the expression of genes involved in antioxidant enzymes such as superoxide dismutase (SOD), catalase (CAT), and glutathione peroxidase (GPx), which can increase the antioxidant capacity of liver cells [134]. In addition, phenolic and flavonoid compounds can inhibit the activation of pro-inflammatory cytokines, such as tumor necrosis factor-alpha (TNF-) and interleukin-6 (IL-6), which can trigger inflammation and liver damage [135,136]. In fact, many countries around the world have taken steps to protect their populations and ecosystems from the dangerous effects of CPF and IM. Therefore, taking into account this scientific knowledge and exercising precaution, the use of CPF and IM has been banned or restricted in different regions.

## 5. Conclusions

IM and CPF were administered daily for 28 days at doses of 14, 28, and 54 mg/kg body weight of IM and 1, 2, and 4 mg/kg body weight of CPF via drinking water. In male rats, IM and CPF caused a reduction in their body weight gain and hepatotoxic effects, as evidenced by increases in the liver enzymes AST, ALT, and ALP. They caused oxidative damage in the liver of male rats as indicated by the decreased liver activity of the GST, GP_X_, SOD, and CAT enzymes and decreased serum TAC. IM and CPF produced a significant dose-dependent increase in DNA damage in hepatocyte cells, resulting in moderate-to-severe liver damage with more cells that are inflammatory, enlarged sinusoids, and compacted nuclei. IM had a higher BMD than that of CPF for both the body and liver weight, suggesting that CPF was more dose-dependently toxic than IM. Albumin was a highly sensitive liver biomarker for the IM-treated rats, while the total protein was a biomarker for the CPF-treated rats. GPx was an extremely sensitive biomarker of oxidative stress in the IM treatment, while CAT and GPx were highly sensitive parameters in the CPF-treated rats. Therefore, at comparable doses, CPF has a higher potential to cause liver damage and oxidative stress than IM. The hepatotoxicity of IM and CPF can be mitigated by administering a nanoemulsion containing clove oil and pomegranate extract. The nanoemulsion acts as a protector against the oxidative stress caused by these insecticides, especially at high doses. The nanoemulsion based on clove oil increases the bioavailability and stability of the pomegranate extract, which has antioxidant properties.

## Figures and Tables

**Table 1:** Weekly body weight gain and relative liver weight of male rats during the CPF or IM treatment experimental period and the ameliorating effect of pomegranate-extract-loaded clove-oil-based nanoemulsion.

Group	Initial bw (g)	Final bw (g)	Weekly bw Gain (g)	% of Weekly bw Gain	Liver Weight	Relative Liver Weight
I	142.00 ± 2.30	220.80 ± 2.60 ^ab^	19.70 ± 0.80 ^a^	100.00	6.88 ± 0.63 ^cd^	3.11 ± 0.26 ^cd^
II	142.80 ± 1.90	220.40 ± 2.40 ^abc^	19.40 ± 1.04 ^ab^	98.48	6.57 ± 0.24 ^d^	2.98 ± 0.12 ^d^
III	145.20 ± 2.20	218.60 ± 1.90 ^bc^	18.35 ± 0.80 ^bc^	93.15	6.89 ± 0.36 ^bcd^	3.15 ± 1.40 ^cd^
IV	146.60 ± 2.10	218.80 ± 2.60 ^bc^	18.05 ± 0.90 ^c^	91.62	7.06 ± 0.43 ^bcd^	3.23 ± 0.21 ^bc^
V	148.20 ± 1.10	218.40 ± 2.30 ^bc^	17.55 ± 0.70 ^cd^	89.09	7.42 ± 0.29 ^ab^	3.39 ± 0.13 ^ab^
VI	149.00 ± 2.40	219.80 ± 1.50 ^abc^	17.70 ± 0.80 ^cd^	89.85	7.08 ± 0.17 ^bcd^	3.22 ± 0.06 ^bcd^
VII	150.40 ± 0.50	217.60 ± 1.10 ^cd^	16.80 ± 0.20 ^de^	85.28	7.39 ± 0.50 ^abc^	3.40 ± 0.24 ^ab^
VIII	149.60 ± 1.90	215.40 ± 1.80 ^d^	16.45 ± 0.90 ^e^	83.50	7.73 ± 0.26 ^a^	3.59 ± 0.10 ^a^
IX	148.00 ± 1.00	222.00 ± 2.10 ^a^	18.50 ± 0.60 ^bc^	93.91	6.92 ± 0.29 ^bcd^	3.12 ± 0.11 ^cd^
X	150.00 ± 1.60	221.80 ± 1.60 ^a^	17.95 ± 0.80 ^c^	91.12	6.71 ± 0.35 ^d^	3.02 ± 0.17 ^cd^

Values are the mean ± S.D. Values with the same letter have insignificant differences at *p* ≤ 0.05. Control group (I); Pomegranate–clove-oil-based nanoemulsion group (II); Imidacloprid-treated groups (III, IV, and V) which received IM in drinking water at doses of 14, 28, and 54 mg/kg bw/day, respectively; Chlorpyrifos-treated groups (VI, VII, and VIII) which received CPF in drinking water at doses of 1, 2, and 4 mg/kg bw/day, respectively. IM pomegranate–clove-oil-based nanoemulsion treatment group (IX) received IM at the highest dose (54 mg/kg bw/day) plus nanoemulsion at a dose of 50 mg/kg bw/day. CPF pomegranate–clove-oil-based nanoemulsion treatment group (X) received CPF at the highest dose (4 mg/kg bw/day) plus nanoemulsion at the dose of 50 mg/kg bw/day. Weekly body weight (g) = (final bw − initial bw)/no. of weeks. Relative liver weight (%) = (liver weight (g)/final bw (g) × 100). Percentage of weekly bw gain (%) = (Weekly bw gain of treatment/Weekly bw gain of control) × 100.

**Table 2:** Effect of IM and CPF on liver function biomarkers and the ameliorative effect of pomegranate-extract-loaded clove-oil-based nanoemulsion.

Group	AST (U/L)	ALT (U/L)	ALP (IU/L)	Total Bilirubin (mg/dL)	Total Protein (g/dL)	Albumin (g/dL)
I	11.55 ± 0.43 ^h^	8.45 ± 0.49 ^f^	38.75 ± 2.39 ^g^	0.56 ± 0.06 ^g^	6.75 ± 0.18 ^h^	3.60 ± 0.09 ^b^
II	9.98 ± 0.58 ^i^	7.27 ± 0.54 ^g^	39.79 ± 2.73 ^g^	0.43 ± 0.06 ^h^	6.96 ± 0.09 ^h^	3.82 ± 0.18 ^a^
III	13.51 ± 0.39 ^g^	10.30 ± 0.54 ^e^	60.32 ± 6.52 ^f^	0.80 ± 0.05 ^f^	8.74 ± 0.12 ^fe^	3.49 ± 0.07 ^b^
IV	17.64 ± 1.58 ^ed^	12.89 ± 1.08 ^c^	76.45 ± 5.13 ^e^	0.98 ± 0.08 ^ed^	9.08 ± 0.19 ^dc^	3.33 ± 0.04 ^c^
V	24.81 ± 2.11 ^b^	15.04 ± 0.75 ^b^	93.62 ± 6.69 ^d^	1.21 ± 0.13 ^b^	9.40 ± 0.17 ^b^	3.17 ± 0.06 ^de^
VI	16.46 ± 0.42 ^fe^	12.86 ± 0.70 ^c^	81.68 ± 7.49 ^e^	0.89 ± 0.05 ^fe^	8.86 ± 0.13 ^ed^	3.26 ± 0.03 ^cd^
VII	21.39 ± 1.50 ^c^	14.37 ± 0.60 ^b^	106.81 ± 7.35 ^c^	1.09 ± 0.08 ^c^	9.17 ± 0.16 ^c^	3.08 ± 0.07 ^e^
VIII	28.00 ± 1.23 ^a^	16.40 ± 0.56 ^a^	178.13 ± 6.85 ^a^	1.50 ± 0.03 ^a^	9.67 ± 0.11 ^a^	2.78 ± 0.21 ^f^
IX	15.26 ± 1.48 ^f^	12.03 ± 0.85 ^dc^	84.40 ± 6.72 ^e^	1.06 ± 0.11 ^dc^	8.58 ± 0.29 ^f^	3.34 ± 0.08 ^c^
X	18.48 ± 0.79 ^d^	11.58 ± 0.95 ^d^	157.50 ± 10.22 ^b^	1.15 ± 0.07 ^cb^	8.29 ± 0.21 ^g^	3.14 ± 0.07 ^de^

Values are the mean ± S.D. Values with the same letter have insignificant differences at *p* ≤ 0.05. Control group (I); Pomegranate–clove-oil-based nanoemulsion group (II); Imidacloprid-treated groups (III, IV, and V) which received IM in drinking water at doses of 14, 28, and 54 mg/kg bw/day, respectively; Chlorpyrifos-treated groups (VI, VII, and VIII) which received CPF in drinking water at doses of 1, 2, and 4 mg/kg bw/day, respectively. IM pomegranate–clove-oil-based nanoemulsion treatment group (IX) received IM at the highest dose (54 mg/kg bw/day) plus nanoemulsion at a dose of 50 mg/kg bw/day. CPF pomegranate–clove-oil-based nanoemulsion treatment group (X) received CPF at the highest dose (4 mg/kg bw/day) plus nanoemulsion at the dose of 50 mg/kg bw/day. (ALP) Alkaline phosphatase, (ALT) alanine aminotransferases, and (AST) aspartate aminotransferases.

**Table 3:** Oxidative stress biomarkers in liver homogenate and serum of male rats exposed to IM and CPF and the ameliorative effect of pomegranate-extract-loaded clove-oil-based nanoemulsion.

Group	Oxidative Stress Markers				
	Liver Tissue				Serum
	GST (U/g. t.)	GPx (U/g. t.)	SOD (U/g. t.)	CAT (U/g. t.)	TAC (mM/L)
I	12.73 ± 0.14 ^b^	217.88 ± 7.7 ^b^	670.2 ± 6.5 ^b^	0.55 ± 0.03 ^b^	1.11 ± 0.02 ^b^
II	12.29 ± 0.15 ^c^	252.89 ± 23.2 ^a^	676.95 ± 19.8 ^a^	0.66 ± 0.02 ^a^	1.15 ± 0.01 ^a^
III	11.91 ± 0.19 ^d^	180.27 ± 12.4 ^dc^	637.61 ± 3.9 ^c^	0.52 ± 0.03 ^cb^	0.98 ± 0.02 ^c^
IV	10.82 ± 0.27 ^f^	172.49 ± 7.4 ^dc^	574.05 ± 11.5 ^e^	0.48 ± 0.02 ^d^	0.85 ± 0.04 ^d^
V	9.75 ± 0.22 ^h^	137.47 ± 5.1 ^e^	521.1 ± 5.2 ^g^	0.38 ± 0.02 ^e^	0.71 ± 0.03 ^f^
VI	13.44 ± 0.13 ^a^	164.71 ± 6.6 ^d^	607.65 ± 9.6 ^d^	0.49 ± 0.01 ^dc^	0.95 ± 0.03 ^c^
VII	11.18 ± 0.10 ^e^	130.98 ± 5.5 ^e^	497.1 ± 12.1 ^h^	0.42 ± 0.07 ^e^	0.78 ± 0.02 ^e^
VIII	7.78 ± 0.12 ^i^	109.71 ± 4.3 ^f^	401.44 ± 5.04 ^i^	0.33 ± 0.03 ^f^	0.66 ± 0.01 ^g^
IX	10.95 ± 0.26 ^fe^	182.86 ± 9.9 ^c^	574.05 ± 11.3 ^e^	0.51 ± 0.02 ^dc^	0.88 ± 0.04 ^d^
X	10.18 ± 0.16 ^g^	169.89 ± 7.5 ^dc^	543.33 ± 11.9 ^f^	0.49 ± 0.01 ^dc^	0.86 ± 0.04 ^d^

Values are the mean ± S.D. Values with the same letter have insignificant differences at *p* ≤ 0.05. Control group (I); Pomegranate–clove-oil-based nanoemulsion group (II); Imidacloprid-treated groups (III, IV, and V) which received IM in drinking water at doses of 14, 28, and 54 mg/kg bw/day, respectively; Chlorpyrifos-treated groups (VI, VII, and VIII) which received CPF in drinking water at doses of 1, 2, and 4 mg/kg bw/day, respectively. IM pomegranate–clove-oil-based nanoemulsion treatment group (IX) received IM at the highest dose (54 mg/kg bw/day) plus nanoemulsion at a dose of 50 mg/kg bw/day. CPF pomegranate–clove-oil-based nanoemulsion treatment group (X) received CPF at the highest dose (4 mg/kg bw/day) plus nanoemulsion at the dose of 50 mg/kg bw/day. GST, glutathione S-transferase; GPx, glutathione peroxidase; SOD, superoxide dismutase; CAT, catalase; and TAC, total antioxidant capacity.

**Table 4:** Score of DNA damage in hepatocyte cells in liver of male rats exposed to IM and CPF and the ameliorative effect of pomegranate-extract-loaded clove-oil-based nano-emulsion.

Group	Cell Number		Comet Class *				Mean ± SEM
	Total Cells	Comet	0	1	2	3	
I	400	39	361	31	8	0	9.75 ± 0.85
II	400	41	359	34	7	0	10.25 ± 0.48
III	400	51	349	32	14	5	12.75 ± 1.11
IV	400	66	334	36	18	12	16.50 ± 1.04
V	400	95	305	40	24	31	23.75 ± 1.44
VI	400	53	347	31	13	9	13.25 ± 1.25
VII	400	72	328	39	17	16	18.00 ± 1.47
VIII	400	97	303	43	20	34	24.25 ± 1.55
IX	400	63	337	28	16	19	15.75 ± 1.65
X	400	66	334	26	15	25	16.50 ± 1.19

Control group (I); nano-emulsion group (II); imidacloprid-treated groups (III, IV, and V) which received IMI in drinking water at doses of 14, 28, and 54 mg/kg bw/day, respectively; chlorpyrifos-treated groups (VI, VII, and VIII) which received CPF in drinking water at doses of 1, 2 and 4 mg/kg bw/day, respectively. IM pomegranate-extract-loaded clove-oil-based nanoemulsion treatment group (IX) received IMI at highest concentration (54 mg/kg bw/day) plus pomegranate-extract-loaded clove-oil-based nanoemulsion. CPF pomegranate-extract-loaded clove-oil-based nanoemulsion treatment group (X) received highest concentration of CPF (4 mg/kg bw/day) and pomegranate-extract-loaded clove-oil-based nano-emulsion. Values for DNA-damaged cells (%) are the mean ± SD, where *n* = 4 and there are 100 examined cells for each animal. * Classes 0, 1, 2, and 3 (no tail, tail length < diameter of nucleus, tail length between 1× and 2× the diameter of nucleus, and tail length > 2× the diameter of nucleus).

**Table 5:** Evaluation of histopathological severity in liver tissue of a male rat exposed to IM and CPF, and the enhancing effect of a clove-oil-based nanoemulsion loaded with pomegranate extract.

Group	Inflammatory Cell Infiltration	Dilated Sinusoids	Degeneration Changes	Pyknotic Nuclei
I	-	-	-	-
II	-	-	-	-
III	+	+	+	+
IV	+	+	+	+
V	++	++	++	++
VI	+	+	+	+
VII	+	+	+	+
VIII	++	++	++	+
IX	+	+	+	+
X	+	+	+	+

Control group (I); nano-emulsion group (II); imidacloprid-treated groups (III, IV, and V) which received IMI in drinking water at doses of 14, 28, and 54 mg/kg bw/day, respectively; chlorpyrifos-treated groups (VI, VII, and VIII) which received CPF in drinking water at doses of 1, 2 and 4 mg/kg bw/day, respectively. IM pomegranate-extract-loaded clove-oil-based nanoemulsion treatment group (IX) received IMI at highest concentration (54 mg/kg bw/day) plus pomegranate-extract-loaded clove-oil-based nanoemulsion. CPF pomegranate-extract-loaded clove-oil-based nanoemulsion treatment group (X) received highest concentration of CPF (4 mg/kg bw/day) and pomegranate-extract-loaded clove-oil-based nano-emulsion. -: Normal; +: Mild; ++: Moderate.

**Table 6:** Benchmark dose (BMD) and upper (BMDU) and lower (BMDL) confidence limits of BMD (CDE) values of different biomarkers of imidacloprid- and chlorpyrifos-treated rats.

Parameters	Imidacloprid (IM)			Chlorpyrifos (CPF)		
	BMD	BMD Confidence Interval		BMD	BMD Confidence Interval	
		Lowest BMDL	Highest BMDU		Lowest BMDL	Highest BMDU
Body weight	5.885	0.0473	26.2	0.09552	0.00113	0.463
Liver weight	42.29	29.1	53.3	1.327	0.133	3.25
Relative liver weight	-	-	-	1.057	0.201	2.49
ALP	1.464	1.18	2.75	0.00968	0.0053	0.0343
ALT	5.309	2.45	9.06	0.001462	0.000219	0.00581
AST	6.866	3.43	10.8	0.1407	0.0556	0.279
Total protein	0.00115	0.00003	0.0138	0.0002361	0.00002	0.00125
Albumin	19.07	12.5	26.8	0.4124	0.154	0.837
Total bilirubin	0.2874	0.0551	0.941	0.01708	0.00659	0.0381
GP_X_	3.192	1.32	6.4	0.2375	0.139	0.409
TAC	6.98	3.71	10.2	0.4723	0.374	0.614
CAT	14.08	8.5	21.6	0.3634	0.121	0.823
GST	11.73	8.89	14.5	1.358	1.26	1.62
SOD	13.79	12.3	15.9	0.7079	0.619	0.815

ALP: alkaline phosphatase, ALT: alanine aminotransferases, AST: aspartate aminotransferase, GST: glutathione S-transferase, GPx: glutathione peroxidase, SOD: superoxide dismutase, CAT: catalase, and TAC: total antioxidant capacity.

## Data Availability

Data will be made available on request.
